# Inverse correlation between longevity and developmental rate among wild *C. elegans* strains

**DOI:** 10.18632/aging.100960

**Published:** 2016-05-10

**Authors:** Yujin Lee, Wooseon Hwang, Juyoung Jung, Sangsoon Park, Josephine Jill T. Cabatbat, Pan-Jun Kim, Seung-Jae V. Lee

**Affiliations:** ^1^ Department of Life Sciences, Pohang University of Science and Technology, Pohang, Gyeongbuk, South Korea; ^2^ School of Interdisciplinary Bioscience and Bioengineering, Pohang University of Science and Technology, Pohang, Gyeongbuk, South Korea; ^3^ Information Technology Convergence Engineering, Pohang University of Science and Technology, Pohang, Gyeongbuk, South Korea; ^4^ Asia Pacific Center for Theoretical Physics, Pohang, Gyeongbuk, South Korea; ^5^ Department of Physics, Pohang University of Science and Technology, Pohang, Gyeongbuk, South Korea

**Keywords:** aging, lifespan, fitness, development, reproduction, C. elegans

## Abstract

Genetic studies using model organisms have shown that many long-lived mutants display impaired fitness, such as reduced fecundity and delayed development. However, in several wild animals, the association between longevity and fitness does not seem to be inevitable. Thus, the relationship between longevity and fitness in wild organisms remains inconclusive. Here, we determined the correlation between lifespan and fitness, developmental rate and brood size, by using 16 wild-derived *C. elegans* strains originated from various geographic areas. We found a negative correlation between lifespan and developmental rate. In contrast, we did not find such negative correlation between longevity and developmental rate among the individuals of *C. elegans* strains. These data imply that polymorphic genetic variants among wild isolates determine resource allocation to longevity and developmental rate.

## INTRODUCTION

Despite extensive research efforts during the past several decades, aging remains as a mysterious process that almost all organisms experience. Many theories have been proposed to explain the causes of aging. The antagonistic pleiotropy theory suggests that genes that cause aging confer beneficial effects in early life but have harmful effects in the later life stages of an organism [1]. The disposable soma theory proposes that somatic cellular damage accumulates during aging at the expense of protecting reproductive systems, which causes organismal aging [2]. A recently proposed hyper-function theory of aging suggests that over-activation of biological processes, which contribute to development and reproduction, leads to aging in adulthood because of hypertrophy-associated pathologies [3-5]. These theories predict that longevity negatively correlates with fitness. In fact, long-lived animals carrying mutations or those that are subjected to dietary restriction tend to display slow development and reduced reproduction (reviewed in [6-8]). In contrast, long-lived wild guppies grow faster and produce more progeny than short-lived ones [9]. Thus, the negative correlation between longevity and fitness does not seem to be inevitable.

*Caenorhabditis elegans* is one of the best-established model organisms for aging research. *C. elegans* is relatively short-lived, genetically tractable, and amenable to track life-historical traits (reviewed in [10, 11]). Studies using an ample repertoire of *C. elegans* mutants, RNAi techniques, and transgenic animals identified aging-regulatory genes, many of which are evolutionarily conserved in other species (reviewed in [10, 12]).

N2, which was isolated from Bristol, UK, has been widely used as a reference wild-type strain for *C. elegans* genetics research. Many other wild *C. elegans* strains from various geographic areas have also been isolated and characterized. Large-scale analysis of single-nucleotide polymorphism (SNP) patterns, phylogeny, and transcriptomic data indicates genetic variations among the wild isolates of *C. elegans* [13-17]. In addition, wild *C. elegans* strains display variable physiologic characteristics such as dauer (hibernation-like larva) formation, copulatory plug formation, lifespan, immunity, fecundity, body length, and food response [18-30]. However, whether longevity and other physiologic phenotypes among wild *C. elegans* strains are correlated is poorly understood.

In this report, we aimed to determine whether there is any correlation between lifespan and fitness among wild *C. elegans* strains. We found that wild *C. elegans* strains with long lifespan displayed a tendency to develop slowly. We further obtained data supporting the possibility that genetic diversity rather than non-genetic variability may underlie the negative correlation between organismal longevity and developmental rate. Thus, wild *C. elegans* may have allocated limited resources to developmental rate and longevity during evolution.

## RESULTS

### Wild *C. elegans* strains display variable life history traits

**Figure 1:**
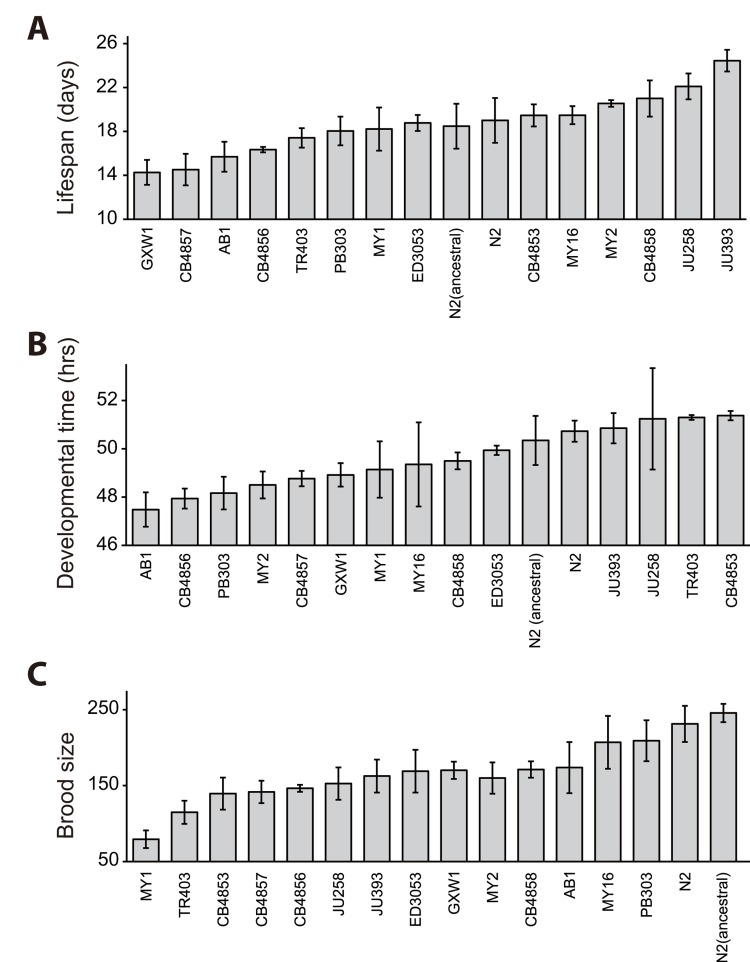
Lifespan, developmental time and total progeny number of wild-derived *C. elegans* strains Lifespan (**A**), developmental time (**B**), and brood size (**C**) of 16 selected wild-derived *C. elegans* strains. Please note that FUdR was used for lifespan assays to prevent progeny hatching. Please also note that the averages of lifespan, developmental time, and brood size of laboratory N2 strain were mostly similar to those of ancestral N2 (*p* > 0.05 for all the comparison, except *p* = 0.004 in one out of three trials of lifespan assays). Please see Table S1 for statistical analysis.

To determine the existence of correlation between lifespan and fitness among wild isolates of *C. elegans*, we extensively measured several life-history traits of 16 wild-derived strains; these were isolated from various places throughout the world (see Materials and Methods). Among the life-history traits, we first measured lifespan, which reflects the degree of aging. We performed lifespan assays in the presence of 5-fluoro-2′-deoxyuridine (FUdR), which is a chemical inhibitor that prevents progeny from hatching by inhibiting DNA synthesis. The average of mean lengths of life of 16 wild *C. elegans* was 18.7 days at 20°C, a standard temperature for *C. elegans* culture in laboratory (Fig. 1A). We first noticed that the mean lifespans significantly varied among strains (Fig. 1A and Table S1; *p* < 0.001, one-way ANOVA-Tukey's comparison test). For example, with FUdR treatment, the mean lifespan of GXW1 was significantly shorter (14.3 days) than that of reference strain N2 (19.0 days), whereas that of JU393 was substantially longer (24.5 days) than that of N2. This variation in lifespan among GXW1, N2 and JU393 was also seen without FUdR treatment (Fig. S1A and B and Table S2).

Next, we measured the time from L1 larvae to adults (developmental time) and the total number of progeny (brood size), which reflect the fitness of a strain. We found that developmental time and brood size varied among strains (Fig. 1B and C and Table S1; *p* = 0.070 for developmental time and *p* < 0.001 for brood size, one way ANOVA-Tukey's comparison test). It is intriguing to note that the average brood sizes of laboratory and ancestral N2 reference strains were larger than those of all the other strains (Fig. 1C; see Discussion for details.). In addition, we found that one strain, RW7000, displayed severely delayed development and semi-sterility phenotypes (Fig. S2A and B), and therefore we excluded RW7000 from further correlation analysis.

### Longevity correlates with slow development among wild *C. elegans* strains

**Figure 2:**
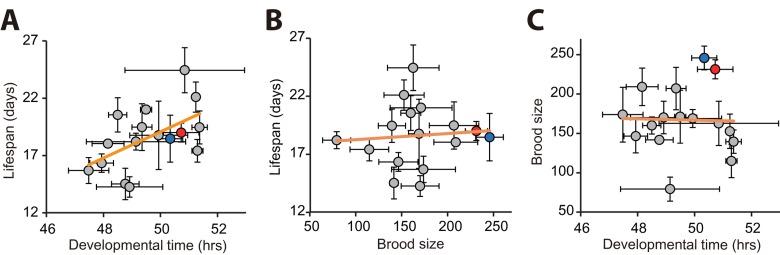
Correlation analysis among developmental time, brood size, and lifespan using populations of wild *C. elegans* strains (**A**) Developmental time correlated with lifespan (*r* = 0.540, *p* = 0.031). (**B**-**C**) In contrast, total progeny number (brood size) did not correlate with lifespan (**B**; *r* = 0.081, *p* = 0.769) or developmental time (**C**; *r* = −0.024, *p* = 0.934). Each circle indicates an average value obtained from independent experiments with populations of each strain. Error bars indicate the standard error of mean (s.e.m.). *r* values are the Pearson correlation coefficients, and their *p* values were calculated by using statistical significance test (see Materials and Methods). Orange lines indicate linear regression lines. Data for our laboratory N2 and ancestral N2 were shown as red and blue circles, respectively. Note that FUdR was used for lifespan assays to prevent progeny hatching. See Table S1 for data values and statistical analysis for each strain.

We analyzed correlations among the three parameters of life-history traits: lifespan (in the presence of FUdR), developmental time and brood size. Importantly, we found a significant correlation between lifespan and developmental time (Fig. 2A; *r* = 0.540, *p* = 0.031). In contrast, we did not find a significant correlation between lifespan and brood size (Fig. 2B; *r* = 0.081, *p* = 0.769). In addition, developmental time did not correlates with brood size (Fig. 2C; *r* = −0.024, *p* = 0.934). Together, these data imply that long lifespan correlates with slow development among wild *C. elegans* strains.

### Individuals of *C. elegans* strains did not display correlation between longevity and developmental rate

**Figure 3:**
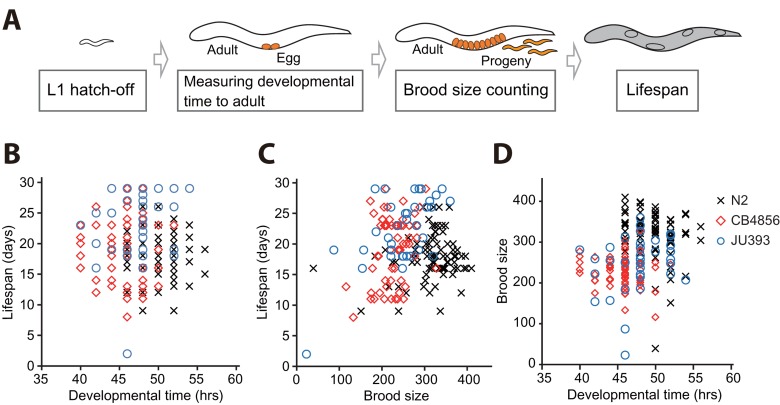
Correlation analysis among developmental time, brood size, and lifespan using individuals of isogenic wild *C. elegans* strains (**A**) A schematic for measuring developmental time from L1 hatchlings to adults, brood size, and adult lifespan of isogenic individual worms. (**B**-**C**) Lifespan and developmental time (**B**; *r* = −0.062, *p* = 0.405) and lifespan and brood size (**C**; *r* = 0.121, *p* = 0.104) did not display a significant correlation. (**D**) Developmental time weakly but significantly correlates with brood size (**D**; *r* = 0.311, *p* < 0.001). Marks (crosses for individuals of N2, diamonds for those of CB4856 and circles for those of JU393) indicate corresponding two parameters of individual worms. *r* values are the Pearson correlation coefficients, and their *p* values were calculated by using statistical significance test (see Materials and Methods). See Fig. S3 for data values for each strain.

Different genetic backgrounds, or non-genetic heterogeneity such as stochastic effects, may underlie the negative correlation between longevity and developmental rate. Stochastic effects may influence different life-history traits among isogenic populations in almost identical environments (reviewed in [12, 31]). We therefore asked whether a correlation between lifespan and developmental rate exists among individuals of isogenic N2, a standard laboratory *C. elegans* strain. To determine the entire life-historical traits of individual worms, we measured the developmental time, brood size and lifespan of each N2 worm (Fig. 3A and see Materials and Methods). We did not observe any correlation among lifespan, developmental time, and brood size (Fig. 3B-D and Fig. S3A, D, and G) of N2 individuals; no correlation between lifespan and total brood size is also consistent with the previous report [32].

In addition to N2, we extended our analysis of the individuals of *C. elegans* to two other strains, CB4856, one of the most genetically distinct strains from N2 [33], and JU393, the longest lived strain in our lifespan analysis. We confirmed the absence of correlation between lifespan and developmental time among the individuals of these strains (Fig. 3B and Fig. S3B-C). Interestingly, the individuals of CB4856 and JU393 displayed a positive correlation between lifespan and brood size (Fig. 3C and Fig. S3E-F; *r* = 0.280, *p* < 0.05 and *r* = 0.619, *p* < 0.001, respectively). These results indicate that long-lived individual worms can also be prolific. In addition, we noticed the absence of a significant correlation between developmental time and brood size among individuals of CB4856 and JU393 strains (Fig. S3H-I). However, when we combined data of individual N2, CB4856 and JU393 strains, developmental time weakly but significantly correlated with brood size (Fig. 3D; r = 0.311, p < 0.001). Nevertheless, our data overall point to the absence of correlation between lifespan and developmental time among individuals of *C. elegans* isolates. Thus, it seems likely that genetic differences among wild-isolate strains are responsible for the negative correlation between lifespan and developmental rate.

## DISCUSSION

Several reports have shown that long-lived *C. elegans* mutants display fitness costs (reviewed in [6-8]). However, studies that examined correlations between longevity and early fitness in wild *C. elegans* are relatively scarce [22, 34]. In this report, we determined the correlation between longevity and fitness among multiple wild *C. elegans* strains from various geographical origins. We showed that worm strains that developed slowly tended to live longer, whereas the total number of progeny did not correlate with longevity. This tendency seems to be attributed to polymorphic variants, as we did not observe the correlation among isogenic individuals. Consistent with our data using *C. elegans*, long-lived wild-isolate mouse strains display a tendency of slow development [35]. Conversely, early maturated female primates display reduced adult survival rate [36]. Several studies using wild vertebrates such as birds, mammals and reptiles have also shown a negative correlation between survival time and age at first reproduction, which is indicative of developmental time (reviewed in [37]). In addition, sexual maturation periods of long-lived species are generally longer than those of short-lived species [38, 39]. Moreover, inhibition of growth correlates with inhibition of senescence in cultured mammalian cells [40, 41]. Thus, our experimental results using wild *C. elegans* strains appear to recapitulate the negative correlation between developmental rate and longevity both among and within species.

**Figure 4:**
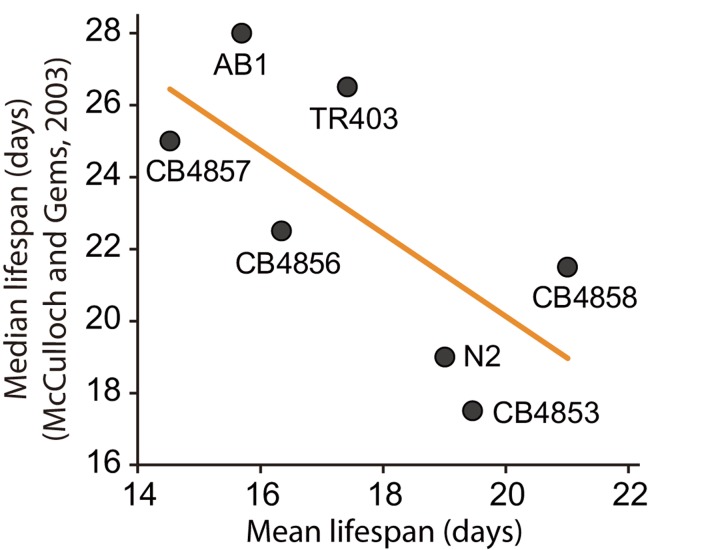
Correlation analysis between lifespan results in previous study (McCulloch and Gems, 2003) and that in this study Shown are mean lifespans (this study) and median lifespans (McCulloch and Gems, 2003) of seven wild *C. elegans* strains that were used in both studies. The lifespans negatively correlated with each other (*r* = 0.686), although the correlation is not significant (*p* = 0.089) perhaps because of small n (n=7). Orange line indicates a linear regression line. Please see Discussion for detailed description.

Several studies have previously investigated the relationship among key life-history traits using wild *C. elegans* strains [22, 34]. McCulloch and Gems showed the absence of correlation between median lifespan and adult body size among 12 wild *C. elegans* strains. We noted that 7 strains were common between their study and ours, and the tendency of lifespans between these two studies seems different from each other (Fig. 4).

Several noticeable differences in experimental conditions exist, including the use of FUdR in our study, culture media (solid vs. liquid) and temperatures (20.0°C vs. 22.5°C) for lifespan assays, developmental rate vs. body length; mean lifespan vs. median lifespan. Thus, these different experimental conditions may have led to differences in life-history traits among the wild *C. elegans* strains. Another study showed the absence of association between longevity and reproduction, as well as longevity and development in heterogeneous wild *C. elegans* populations during experimental evolution in laboratory [34]. The experimental design of their study is suitable for examining a negative correlation between lifespan and fitness. Specifically, they used ancestral and evolved lines of heterogeneous wild *C. elegans* strains, which were generated by mating multiple wild *C. elegans* strains reciprocally to represent the diversity of wild *C. elegans*. Their experimental setup enriched strains with increased early reproduction, which is likely to have selected strains with fast development. This may have negatively selected strains with long lifespan. It will be interesting to determine the correlation between lifespan and developmental rate among and within ancestral and evolved isogenic wild isolates at the same time in future studies.

The ecology of specific *C. elegans* strains may affect various traits measured in laboratory experiments. For example, the life-history traits that we examined may have been affected by the environmental temperatures, to which the strains are adjusted in their natural habitats. As an attempt to address this possibility, we analyzed the correlation between average annual temperatures of the regions that the strains were isolated (Table S3) and the life-history traits that we measured. We did not find any correlation among the strains that we used (Fig. S4A-C). A recent paper has shown that regions of isolation had no effect on differences in fertility among 22 wild *C. elegans* strains, which is consistent with our results [42]. It will be interesting to examine the relationship between the ecology and life history traits further with more wild *C. elegans* strains under various environmental conditions in the future.

Although our study provides rich information regarding life-history traits of various wild *C. elegans* strains, a number of limitations still exist. First, a negative correlation between developmental rate and lifespan does not directly infer a trade-off. This is because our current study lacks data supporting a causal relationship for the trade-off. This should be done in future research using molecular genomic and genetic approaches. Second, the number of strains that we examined covers only a small portion of over 200 wild *C. elegans* strains that have been isolated so far [43]. Third, genetic variations across wild *C. elegans* strains are relatively low, even among wild isolates that have originated from diverse geographic regions [13, 43-45]. Therefore, it is possible that multiple linked alleles, not a pleiotropic allele, may have affected various traits such as lifespan and development by chance. Fourth, our experimental conditions are likely to be optimized for the fitness of N2, a laboratory reference strain. This may have skewed experimental data for various other wild strains (reviewed in [46]). In fact, our data are consistent with this possibility, because the total progeny number of N2 was the largest among the wild *C. elegans* strains that we used. It is also possible that laboratory conditions may have selected a highly fecund sub-strain of N2 [47]. Fifth, our experimental setup is far from natural environments that *C. elegans* strains live in. For example, although we obtained consistent lifespan results with or without FUdR treatment among three selected strains, our lifespan analysis for a majority of strains was performed with FUdR. Additionally, environmental factors such as the presence of pathogens and temperature are different from those of nature. This may have further differentiated our experimental conditions from natural environments. Thus, future research using more natural experimental settings, such as lifespan assays without FUdR, is required. Nevertheless, we believe our study provides valuable information to the field of aging research, because this is one of the first studies that extensively measured correlations among crucial life-history traits in wild *C. elegans* strains.

One of the most recent and influential theories of aging is the hyperfunction theory, also known as the quasi-programmed theory of aging (reviewed in [3-5, 48]). The hyperfunction theory of aging suggests that aging is a continuation of developmental growth, and hyperactivation of developmental programs during post-developmental periods leads to pathologies and aging (reviewed in [3-5, 48]). Our key data showing that fast developing wild *C. elegans* isolates tend to age quickly support the hyperfunction theory of aging. At the molecular level, continuous activation of target of rapamycin (TOR) signaling, whose inhibition delays both development and aging in many species (reviewed in [49]), has been suggested as the basis of the quasi-programmed aging [50, 51]. Thus, it will be worth examining whether the activities of TOR signaling among wild *C. elegans* isolates correlate with developmental rate and/or lifespan in future research.

## MATERIALS AND METHODS

### Nematode strains and maintenance

*C. elegans* strains that originated from various geographic regions (Table S3) were obtained from Caenorhabditis Genetics Center, which is funded by the NIH National Center for Research Resources: ancestral N2 (Bristol, UK), AB1 (Adelaide, Australia), CB4853 (Altadena, USA), CB4856 (Hawaii, USA), CB4857 (Claremont, USA), CB4858 (Pasadena, USA), ED3053 (Limuru, Kenya), GXW1 (Wuhan, China), JU258 (Madeira, Portugal), JU393 (Hermanville, France), MY1 (Lingen, Germany), MY2 (Münster, Germany), MY16 (Münster, Germany), N2 (the reference strain, Bristol, UK), PB303 (unknown geographic origin, USA), RW7000 (Bergerac, France), TR403 (Madison, USA). The origin of ancestral N2 strain is from one of the earliest frozen stocks of N2. All strains were maintained on NGM plates seeded with *Escherichia coli* OP50 strain as a food source at 20°C. Reference N2 strain was included as a control for all the experiments.

### Lifespan assays

Lifespan assays were performed as previously described with minor modifications [54]. Gravid worms were allowed to lay eggs for 12 hours to synchronize progeny. When progeny reached young adult stages, approximately 100 worms were transferred onto four NGM plates containing 5 μM of 5-fluoro-2′-deoxyuridine (FUdR, Sigma, St. Louis, MO, USA). For the experiment shown in Fig. S1, approximately 150 worms were transferred onto six fresh NGM plates without FUdR every 1-2 days until worms did not lay progeny. All lifespan assays were conducted independently at 20°C at least twice. OASIS (online application for the survival analysis, http://sbi.postech.ac.kr/oasis) was used for statistical analysis [55]. Worms that were missing, burrowed, crawled off, or displayed internal hatching or vulval protrusion were censored but included in the analysis. Data were analyzed by using one-way ANOVA and Tukey's multiple comparison test functions in GraphPad Prism 6 software (free trial version).

### Measurement of brood size

Total brood size measurement was performed as previously described with minor modifications [54, 56]. Each L4 stage hermaphrodite was transferred to a fresh NGM plate every 24 hours for four days. The brood size of each worm was the total number of hatched progeny during the duration of the assay. Unhatched eggs were not counted as viable progeny. Adult worms that were missing, burrowed, crawled off, dead, or displayed internal hatching or protruding vulvae were excluded from the analysis. All brood size assays were performed at least three times independently at 20°C. Data were analyzed by using one-way ANOVA and Tukey's multiple comparison tests in GraphPad Prism 6 software (free trial version).

### Measurement of developmental time

Developmental assay was performed as previously described with minor modifications [57]. Worms were washed off replete NGM plates with M9 buffer, and remaining eggs were incubated at 20°C for 1-2 hours for synchronization. Forty newly-hatched L1 progeny were transferred to new OP50-seeded NGM plates and were allowed to develop. Worms that have at least one egg in their bodies were considered as adults. The number of adult worms was counted every 2 hours after 40 hours from the L1 transfer. The developmental time of all 16 wild *C. elegans* strains were simultaneously measured twice independently at 20°C. OASIS (online application for the survival analysis, http://sbi.postech.ac.kr/oasis) was used for statistical analysis [55]. Data were analyzed by using one-way ANOVA and Tukey's multiple comparison test functions in GraphPad Prism 6 software (free trial version).

### Measurement of brood size, developmental rate, and lifespan of isogenic individuals

Adult worms were washed off replete NGM plates with M9 buffer, and remaining eggs were allowed to hatch at 20°C for 1 hour for synchronization. Newly-hatched individual L1 worms were transferred to new individual OP50-seeded NGM plates, and allowed to develop. Worms that have at least one egg in their bodies were considered as adults. The developmental time for each worm was recorded every 2 hours until the worm reached adulthood. The adult worms were then transferred to new individual NGM plates every 12 hours until they did not lay eggs. The brood size of each worm was the total number of hatched progeny during the duration of the assay. Worms that ceased producing eggs were transferred to new individual NGM plates for continued lifespan assays. Worms were considered as alive if they moved when prodded. All assays were conducted at 20°C three times independently. OASIS (online application for the survival analysis, http://sbi.postech.ac.kr/oasis) was used for statistical analysis for lifespan and developmental time [55]. Worms that were missing, burrowed, crawled off, or displayed internal hatching or sterility were excluded from the analysis. Although more than 100 individuals of N2, CB4856 and JU393 were used for initial experiments, only 82, 64, and 35 individuals remained respectively for analysis due to censoring during performing the assays.

### Calculation of *p* values for the Pearson correlation coefficient *r*

A web-based calculator was used to obtain *p* values (http://www.socscistatistics.com/) for correlations in this study. The obtained *p* values for several datasets were also manually confirmed as follows. The statistical significance of the Pearson correlation coefficient *r* between two variables *x_i_* and *y_i_* (*i* = 1, 2, ···, *N*) was tested under the null hypothesis that there is no association between *x_i_* and *y_i_*. We numerically obtained the null distribution of *r*, when *x_i_*'s (*y_i_*'s) were randomly permuted against *y_i_*'s (*x_i_*'s). From this null distribution, two-sided *p* values were computed.

## SUPPLEMENTAL DATA



## References

[1] Williams GC (1957). Pleiotropy, Natural Selection, and the Evolution of Senescence. Evolution.

[2] Kirkwood TB (1977). Evolution of ageing. Nature.

[3] Blagosklonny MV (2013). MTOR-driven quasi-programmed aging as a disposable soma theory: blind watchmaker vs. intelligent designer. Cell Cycle.

[4] Blagosklonny MV (2013). Aging is not programmed: genetic pseudo-program is a shadow of developmental growth. Cell cycle.

[5] Gems D, de la Guardia Y (2013). Alternative perspectives on aging in *Caenorhabditis elegans*: reactive oxygen species or hyperfunction?. Antioxidants & redox signaling.

[6] Antebi A (2007). Genetics of aging in Caenorhabditis elegans. PLoS Genetics.

[7] Partridge L, Gems D (2006). Beyond the evolutionary theory of ageing, from functional genomics to evo-gero. Trends in Ecology & Evolution.

[8] Hekimi S (2006). How genetic analysis tests theories of animal aging. Nature Genetics.

[9] Reznick DN, Bryant MJ, Roff D, Ghalambor CK, Ghalambor DE (2004). Effect of extrinsic mortality on the evolution of senescence in guppies. Nature.

[10] Lapierre LR, Hansen M (2012). Lessons from *C. elegans*: signaling pathways for longevity. Trends in Endocrinology and Metabolism: TEM.

[11] Tissenbaum HA (2015). Using for aging research. Invertebrate reproduction & development.

[12] Kenyon CJ (2010). The genetics of ageing. Nature.

[13] Koch R, van Luenen HG, van der Horst M, Thijssen KL, Plasterk RH (2000). Single nucleotide polymorphisms in wild isolates of Caenorhabditis elegans. Genome Research.

[14] Denver DR, Morris K, Thomas WK (2003). Phylogenetics in Caenorhabditis elegans: an analysis of divergence and outcrossing. Molecular Biology and Evolution.

[15] Volkers RJ, Snoek LB, Hubar CJ, Coopman R, Chen W, Yang W, Sterken MG, Schulenburg H, Braeckman BP, Kammenga JE (2013). Gene-environment and protein-degradation signatures characterize genomic and phenotypic diversity in wild Caenorhabditis elegans populations. BMC Biology.

[16] Vu V, Verster AJ, Schertzberg M, Chuluunbaatar T, Spensley M, Pajkic D, Traver Hart G, Moffat J, Fraser AG (2015). Natural variation in gene expression modulates the severity of mutant phenotypes. Cell.

[17] Thompson O, Edgley M, Strasbourger P, Flibotte S, Ewing B, Adair R, Au, Chaudhry I, Fernando L, Hutter H, Kieffer A, Lau J, Lee N, Miller A, Raymant G, Shen B, Shendure J, Taylor J, Turner EH, Hillier LW, Moerman DG, Waterston RH (2013). The million mutation project: a new approach to genetics in *Caenorhabditis elegans*. Genome Research.

[18] Hodgkin J, Doniach T (1997). Natural variation and copulatory plug formation in *Caenorhabditis elegans*. Genetics.

[19] de Bono M, Bargmann CI (1998). Natural variation in a neuropeptide Y receptor homolog modifies social behavior and food response in C. elegans. Cell.

[20] Gems D, Riddle DL (2000). Defining wild-type life span in *Caenorhabditis elegans*. The journals of gerontology Series A, Biological Sciences and Medical Sciences.

[21] Knight CG, Azevedo RB, Leroi AM (2001). Testing life-history pleiotropy in *Caenorhabditis elegans*. Evolution.

[22] McCulloch D, Gems D (2003). Body size, insulin/IGF signaling and aging in the nematode *Caenorhabditis elegans*. Experimental Gerontology.

[23] McCulloch D, Gems D (2003). Evolution of male longevity bias in nematodes. Aging Cell.

[24] Viney ME, Gardner MP, Jackson JA (2003). Variation in *Caenorhabditis elegans* dauer larva formation. Development, Growth & Differentiation.

[25] Harvey SC, Shorto A, Viney ME (2008). Quantitative genetic analysis of life-history traits of *Caenorhabditis elegans* in stressful environments. BMC Evolutionary Biology.

[26] Green JW, Snoek LB, Kammenga JE, Harvey SC (2013). Genetic mapping of variation in dauer larvae development in growing populations of *Caenorhabditis elegans*. Heredity.

[27] Sutphin GL, Kaeberlein M (2008). Dietary restriction by bacterial deprivation increases life span in wild-derived nematodes. Experimental Gerontology.

[28] Reddy KC, Andersen EC, Kruglyak L, Kim DH (2009). A polymorphism in npr-1 is a behavioral determinant of pathogen susceptibility in *C. elegans*. Science.

[29] Balla KM, Andersen EC, Kruglyak L, Troemel ER (2015). A Wild *C. Elegans* Strain Has Enhanced Epithelial Immunity to a Natural Microsporidian Parasite. PLoS Pathog.

[30] Stastna JJ, Snoek LB, Kammenga JE, Harvey SC (2015). Genotype-dependent lifespan effects in peptone deprived *Caenorhabditis elegans*. Scientific Reports.

[31] Kirkwood TB, Feder M, Finch CE, Franceschi C, Globerson A, Klingenberg CP, LaMarco K, Omholt S, Westendorp RG (2005). What accounts for the wide variation in life span of genetically identical organisms reared in a constant environment?. Mechanisms of Ageing and Development.

[32] Chen J, Senturk D, Wang JL, Muller HG, Carey JR, Caswell H, Caswell-Chen EP (2007). A demographic analysis of the fitness cost of extended longevity in *Caenorhabditis elegans*. The Journals of Gerontology Series A, Biological Sciences and Medical Sciences.

[33] Thompson OA, Snoek LB, Nijveen H, Sterken MG, Volkers RJ, Brenchley R, Hof A, Bevers RPJ, Cossins AR, Yanai I, Hajnal A, Schmid T, Perkins JD, Spencer D, Kruglyak L, Andersen EC, Moerman DG, Hillier LW, Kammenga JE, Waterston RH (2015). Remarkably divergent regions punctuate the genome assembly of the *Caenorhabditis elegans* Hawaiian strain CB4856. Genetics.

[34] Anderson JL, Reynolds RM, Morran LT, Tolman-Thompson J, Phillips PC (2011). Experimental evolution reveals antagonistic pleiotropy in reproductive timing but not life span in *Caenorhabditis elegans*. The Journals of Gerontology Series A, Biological Sciences and Medical sciences.

[35] Miller RA, Harper JM, Dysko RC, Durkee SJ, Austad SN (2002). Longer life spans and delayed maturation in wild-derived mice. Experimental Biology and Medicine (Maywood, NJ).

[36] Blomquist GE (2009). Trade-off between age of first reproduction and survival in a female primate. Biology Letters.

[37] Lemaitre JF, Berger V, Bonenfant C, Douhard M, Gamelon M, Plard F, Gaillard JM (2015). Early-late life trade-offs and the evolution of ageing in the wild. Proceedings Biological Sciences / The Royal Society.

[38] Ricklefs RE (2010). Life-history connections to rates of aging in terrestrial vertebrates. Proceedings of the National Academy of Sciences of the United States of America.

[39] Fushan AA, Turanov AA, Lee SG, Kim EB, Lobanov AV, Yim SH, Buffenstein R, Lee SR, Chang KT, Rhee H, Kim JS, Yang KS, Gladyshev VN (2015). Gene expression defines natural changes in mammalian lifespan. Aging Cell.

[40] Leontieva OV, Demidenko ZN, Blagosklonny MV (2015). Dual mTORC1/C2 inhibitors suppress cellular geroconversion (a senescence program). Oncotarget.

[41] Blagosklonny MV (2014). Geroconversion: irreversible step to cellular senescence. Cell cycle.

[42] Petrella LN (2014). Natural variants of *C. elegans* demonstrate defects in both sperm function and oogenesis at elevated temperatures. PloS ONE.

[43] Andersen EC, Gerke JP, Shapiro JA, Crissman JR, Ghosh R, Bloom JS, Felix MA, Kruglyak L (2012). Chromosome-scale selective sweeps shape *Caenorhabditis elegans* genomic diversity. Nature Genetics.

[44] Sivasundar A, Hey J (2003). Population genetics of *Caenorhabditis elegans*: the paradox of low polymorphism in a widespread species. Genetics.

[45] Cutter AD (2006). Nucleotide polymorphism and linkage disequilibrium in wild populations of the partial selfer *Caenorhabditis elegans*. Genetics.

[46] Sterken MG, Snoek LB, Kammenga JE, Andersen EC (2015). The laboratory domestication of *Caenorhabditis elegans*. Trends in Genetics : TIG.

[47] Harvey SC, Viney ME (2007). Thermal variation reveals natural variation between isolates of *Caenorhabditis elegans*. Journal of Experimental Zoology Part B, Molecular and Developmental Evolution.

[48] Blagosklonny MV, Hall MN (2009). Growth and aging: a common molecular mechanism. Aging.

[49] Johnson SC, Rabinovitch PS, Kaeberlein M (2013). mTOR is a key modulator of ageing and age-related disease. Nature.

[50] Leontieva OV, Paszkiewicz GM, Blagosklonny MV (2012). Mechanistic or mammalian target of rapamycin (mTOR) may determine robustness in young male mice at the cost of accelerated aging. Aging (Albany NY).

[51] Scialò F, Sriram A, Naudí A, Ayala V, Jové M, Pamplona R, Sanz A (2015). Target of rapamycin activation predicts lifespan in fruit flies. Cell Cycle.

[52] Hwang W, Artan M, Seo M, Lee D, Nam HG, Lee SV (2015). Inhibition of elongin C promotes longevity and protein homeostasis via HIF-1 in *C. elegans*. Aging Cell.

[53] Yang JS, Nam HJ, Seo M, Han SK, Choi Y, Nam HG, Lee SJ, Kim S (2011). OASIS: online application for the survival analysis of lifespan assays performed in aging research. PloS ONE.

[54] Kim YI, Bandyopadhyay J, Cho I, Lee J, Park DH, Cho JH (2014). Nucleolar GTPase NOG-1 regulates development, fat storage, and longevity through insulin/IGF signaling in *C. elegans*. Molecules and Cells.

[55] Lee SJ, Hwang AB, Kenyon C (2010). Inhibition of respiration extends *C. elegans* life span via reactive oxygen species that increase HIF-1 activity. Current Biology.

