# Supplementary material for: Inverse correlation between longevity and developmental rate among wild C. elegans strains
Source: Aging (Albany NY). 2016 May 10;8(5):986–94. doi: 10.18632/aging.100960 (PMC4931849; doi:10.18632/aging.100960)
Supplement: Supplementary file 1 [file aging-08-0986-s001.pdf]

SUPPLEMENTAL DATA

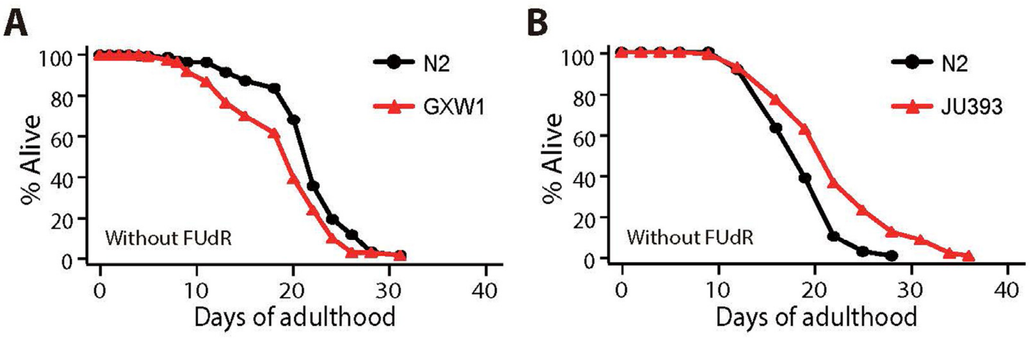

Figure S1. Lifespan of N2, GXW1 and JU393 without FUdR treatment. (**A**) Lifespan of GXW1, which displayed the shortest mean lifespan among wild *C. elegans* strains upon FUdR treatment (Fig. 1A), was shorter than N2 without FUdR treatment (2 out of 3 trials). (**B**) JU393, which displayed the longest mean lifespan among wild *C. elegans* upon FUdR treatment (Fig. 1A), lived longer than N2 without FUdR treatment (2 out of 2 trials). See Table S2 for statistical analysis.

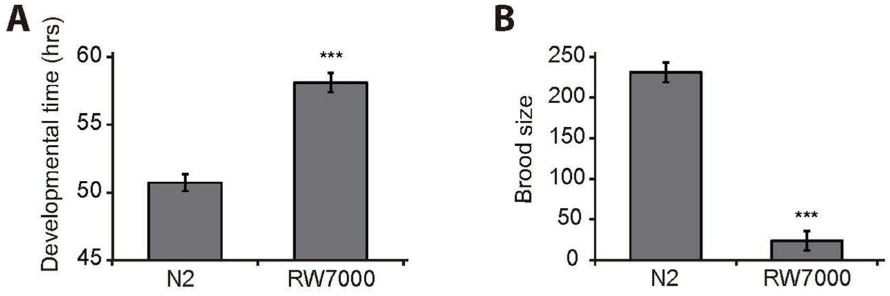

Figure S2. RW7000 displays slow development and semi-sterility. The developmental time (**A**) (n = 67) and the brood size (**B**) (n = 8) of RW7000 were compared with N2 at least twice independently. Error bars represent standard error of mean (s.e.m.) (two-tailed Student's *t*-test, ****p* < 0.001).

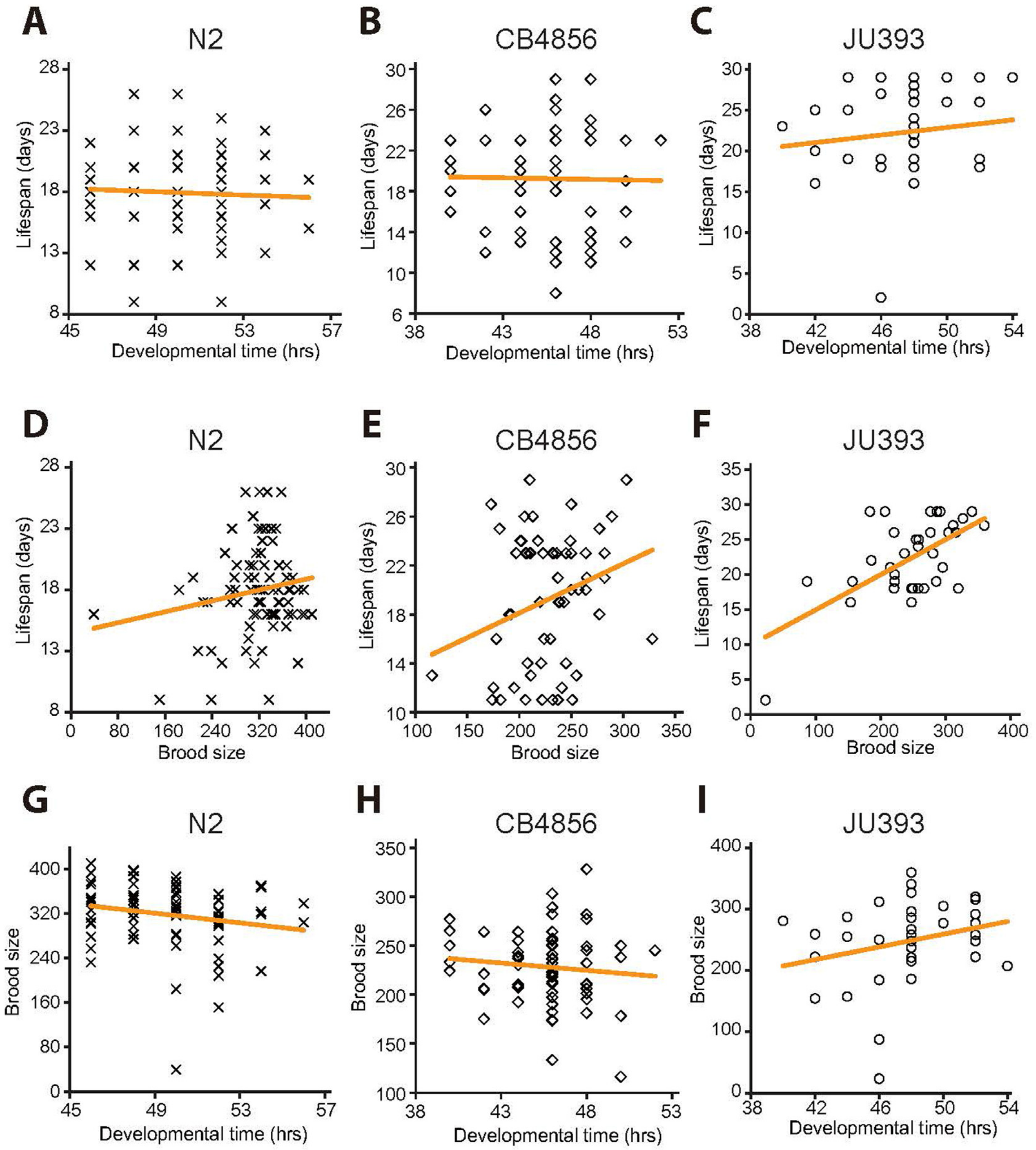

Figure S3. Correlation analysis among developmental time, brood size, and lifespan using individuals of isogenic N2, CB4856, and JU393 strains. (**A**-**C**) Lifespan and developmental time among individuals of N2 (**A**; r = -0.047, p = 0.675), CB4856 (**B**; r = -0.015, p = 0.907), or JU393 (**C**; r = 0.141, p = 0.420) did not display a significant correlation. (**D**-**F**) Lifespan did not correlate with brood size among individuals of N2 (**D**; r = 0.176, p = 0.114), whereas lifespan correlated with brood size among individuals of CB4856 (**E**; r = 0.280, p = 0.025) and JU393 (**F**; r = 0.619, p < 0.001). (**G**-**I**) Developmental time and brood size among individuals of N2 (**G**; r = -0.194, p = 0.081), CB4856 (**H**; r = -0.106, p = 0.407), or JU393 (**I**; r = 0.256, p = 0.138) did not display a significant correlation.

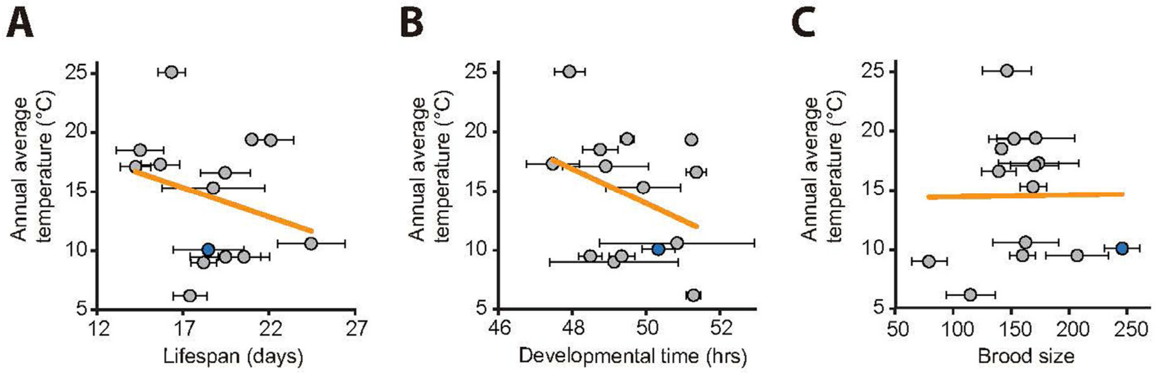

Figure S4. Correlation analysis among mean lifespan, developmental time, brood size and average annual temperatures of the regions where wild *C. elegans* strains originated. Average annual temperature of each strain's origin did not correlate with mean lifespan (**A**; r = -0.271, p = 0.330), developmental time (**B**; r =-0.340, p = 0.215), or brood size (**C**; r = 0.010, p = 0.972). Error bars indicate the standard error of mean (s.e.m.). r values are the Pearson correlation coefficients, and their p values were calculated by using statistical significance test (see Materials and Methods). Orange lines indicate linear regression lines. Data for ancestral N2 were shown as blue circles. See Table S3 for data values and statistical analysis for each strain.

Table S1. Analysis of mean lifespan, developmental time, and brood size of the wild strains examined in this study

Strain	Mean lifespan ±s.e.m. (days)	Sum of animals that died/total (number of trials)	Mean develop-mental time ±s.e.m. (hrs)	Sum of animals that reached adults/ total (number of trials)	Mean brood size±s.e.m.	Total number of tested animals (number of trials)
AB1	15.7±1.1	261/325 (3)	47.5±0.7	75/80 (2)	173.8±34.8	17 (3)
CB4853	19.5±1.4	226/296 (3)	51.4±0.3	79/80 (2)	139.5±14.8	20 (3)
CB4856	16.3±0.8	266/325 (3)	47.9±0.4	53/80 (2)	146.5±21.3	22 (3)
CB4857	14.5±1.4	283/317 (3)	48.8±0.5	58/80 (2)	141.7±4.5	21 (3)
CB4858	21.0±0.2	191/296 (3)	49.5±0.2	78/80 (2)	171.3±33.6	13 (3)
ED3053	18.8±3.0	214/360 (3)	49.9±1.0	66/80 (2)	169.0±11.4	13 (3)
GXW1	14.3±0.9	265/327 (3)	48.9±1.2	80/80 (2)	170.2±20.6	23 (3)
JU258	22.1±1.3	201/295 (3)	51.2±0.1	75/80 (2)	152.7±21.8	16 (3)
JU393	24.5±2.0	262/420 (4)	50.9±2.1	80/80 (2)	162.7±28.1	18 (3)
MY1	18.2±0.7	209/285 (3)	49.1±1.7	77/80 (2)	79.3±15.3	19 (3)

MY2	20.6±1.5	198/360 (3)	48.5±0.3	78/80 (2)	160.0±10.8	12 (3)
MY16	19.5±2.0	130/220 (2)	49.4±0.4	79/80 (2)	207.1±26.9	20 (3)
N2	19.0±0.8	968/1160 (11)	50.7±0.6	80/80 (2)	231.4±12.1	69 (10)
N2*	18.5±2.0	224/300 (3)	50.3±0.4	80/80 (2)	245.8±15.0	20 (3)
PB303	18.0±0.3	134/200 (2)	48.2±0.6	77/80 (2)	209.1±23.9	14 (3)
TR403	17.4±1.0	269/310 (3)	51.3±0.2	77/80 (2)	114.9±21.1	23 (3)

N2*: ancestral N2

Table S2. Lifespan analysis of N2, GXW1 and JU393 strains without FUdR treatment

**Strain**	**Mean lifespan ±s.e.m. (days)**	**75th percentile**	**% change**	**Number of animals that died/total**	***p* value vs. N2**
N2	14.4±0.5	17		68/150	
GXW1	12.5±0.6	17	-13%	54/180	0.0133
N2	21.6±0.4	24		127/180	
GXW1	18.8±0.6	22	-13%	74/150	0.0001
JU393	24.0±0.4	26	+11%	91/150	0.0001
N2	18.3±0.4	22		115/180	
GXW1	18.4±0.5	20	+1%	94/150	0.8901
JU393	24.5±0.7	28	+34%	71/150	<0.0001

Table S3. Summary of information regarding wild *C. elegans* isolates that were tested in this study

Isolate	Location of origin	Latitude	Average summer temperature (°C, High, Low)	Average winter temperature (°C, High, Low)
AB1	Adelaide, Australia	34° 93'S	28, 18	14, 8
CB4853	Altadena, USA	34° 11'N	29.9, 14.3	17.9, 5.7
CB4856	Hawaii, USA	21° 33'N	27.3, 19.1	24.2, 15.5
CB4857	Claremont, USA	34° 07'N	32.2, 16.7	20.0, 6.1
CB4858	Pasadena, USA	34° 09'N	31.4, 15.5	19.1, 5.8
ED3053	Limuru, Kenya	1° 05'S	25, 12	21, 11
GXW1	Wuhan, China	30° 37'N	32, 25	7, 0
JU258	Ribeiro Frio, Portugal	32° 43'N	24, 19	18, 14
JU393	Hermanville, France	49° 17'N	22, 13	7, 2
MY1	Lingen, Germany	52° 54'N	24, 13	6, 0
MY16	Mecklenbeck, Germany	51° 56'N	24, 13	6, 0
MY2	Roxel, Germany	51° 96'N	24, 13	6, 0
N2	Bristol, UK	51° 28'N	22, 14	8, 4
N2 ancestral	Bristol, UK	51° 28'N	22, 14	8, 4
TR403	Madison, USA	43° 04'N	29, 16	-1, -11

Note that the reference N2 strain, which may have been adapted to laboratory conditions, was excluded from correlation analysis between the annual average temperatures of strains' regional origins and the life-history traits (mean lifespan, developmental time and brood size). We also excluded PB303, which does not have specific information about regional origin. Summer temperature represents the average temperature of January (Southern hemisphere) and July (Northern hemisphere), respectively. Conversely, winter temperature represents the average temparature of July (Southern hemisphere) and January (Northern hemisphere).
